# Temperature activated transient receptor potential ion channels from Antarctic fishes

**DOI:** 10.1098/rsob.230215

**Published:** 2023-10-18

**Authors:** Julia M. York

**Affiliations:** ^1^ Department of Integrative Biology, Institute for Neuroscience, University of Texas at Austin, Austin, TX, USA; ^2^ School of Integrative Biology, University of Illinois Urbana–Champaign, Urbana, Illinois, USA

**Keywords:** notothenioids, TRP channels, thermosensation, ion channels, Antarctica

## Abstract

Antarctic notothenioid fishes (cryonotothenioids) live in waters that range between −1.86°C and an extreme maximum +4°C. Evidence suggests these fish sense temperature peripherally, but the molecular mechanism of temperature sensation in unknown. Previous work identified transient receptor potential (TRP) channels TRPA1b, TRPM4 and TRPV1a as the top candidates for temperature sensors. Here, cryonotothenioid TRPA1b and TRPV1a are characterized using *Xenopus* oocyte electrophysiology. TRPA1b and TRPV1a showed heat-evoked currents with Q10s of 11.1 ± 2.2 and 20.5 ± 2.4, respectively. Unexpectedly, heat activation occurred at a threshold of 22.9 ± 1.3°C for TRPA1b and 32.1 ± 0.6°C for TRPV1a. These fish have not experienced such temperatures for at least 15 Myr. Either (1) another molecular mechanism underlies temperature sensation, (2) these fishes do not sense temperatures below these thresholds despite having lethal limits as low as 5°C, or (3) native cellular conditions modify the TRP channels to function at relevant temperatures. The effects of osmolytes, pH, oxidation, phosphorylation, lipids and accessory proteins were tested. No conditions shifted the activity range of TRPV1a. Oxidation in combination with reduced cholesterol significantly dropped activation threshold of TRPA1b to 11.3 ± 2.3°C, it is hypothesized the effect may be due to lipid raft disruption.

## Graphical abstract

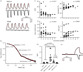

## 1. Introduction

Sensing temperature is vital for organisms because temperature affects most biological systems and physiological processes, including the diffusion of ions across ion channel pores, with a two- to threefold difference in rate for every 10°C change in temperature (Q10 of 2 to 3). Molecular mechanisms of temperature sensation have a greater dependence of activity on temperature and are defined as having Q10 values > 3 [1]. Transient receptor potential (TRP) channels are the best understood molecular thermosensors; TRP channels are membrane integral proteins that convert temperature and other stimuli into electrical signals by opening and closing (or gating) a transmembrane pore to allow the passage of cations into and out of the cell. This can be measured as a flow of current across the cell membrane. There are 10 subfamilies of TRP channels, of which seven are found in metazoans, and temperature activated TRP channels have been found in TRPA, TRPC, TRPM and TRPV subfamilies in vertebrates [2]. Depending on the channel, TRP channels can be heat or cold activated and their activation threshold usually can be correlated with habitat temperature or specific biological functions of the channel (e.g. [3,4]).

Although TRP channels are well-established as a mechanism of noxious heat and mild cool sensation in mammals, these channels are over 1.5 billion years old and their general role in thermosensation in vertebrates is unclear [5]. As variability in the thermal environment on Earth accelerates rapidly with anthropogenic climate change, it becomes more critical that we understand how organisms select their thermal environments and how that process evolves. This is particularly important for marine ectotherms, not only because they determine body temperature through behavioural thermoregulation, but moreover because the ocean absorbs 90% of the heat created by increasing concentrations of greenhouse gases so they are more vulnerable to warming [6,7]. Studying marine regions at the poles is particularly useful as they exemplify future conditions for the rest of the world due to amplified rates of warming [8]. While polar regions may seem remote and unusual, in reality most of the biosphere is constantly below 10°C [9]. Yet, in the study of molecular mechanisms, we have not identified a thermosensitive channel that gates below 8°C and so we do not understand how organisms discriminate most thermal environments.

Temperature-dependent TRP channel gating, although not completely understood, is thought to be associated with a large change in enthalpy or a difference in heat capacity between different channel states [10,11]. For the activation energy of channel opening to be sufficiently low, the large change in enthalpy must be balanced by a large change in entropy. However, cold adapted proteins are characterized by low heat capacity and enthalpy [12–14]. If heat capacity and enthalpy are inherently limited in cold adapted proteins, how can robust temperature dependent gating occur at Antarctic temperatures?

Together, these motivations led to the investigation of TRP channels in a fascinating group of polar ectotherms: the Antarctic notothenioid fishes or cryonotothenioids. Cryonotothenioid fishes are the primary fish fauna of the Southern Ocean which surrounds the Antarctic continent and are an example of an evolutionary radiation with one of the highest rates of speciation in marine teleosts [15,16]. The Southern Ocean is highly stenothermal, ranging generally between −1.9 and +1°C with temperature maxima around +4°C [17,18]. It is estimated that the Southern Ocean has warmed an average of 1°C over the last 50 years, but this warming is spatially heterogeneous [19–21]. Thus, cryonotothenioids are an excellent model both for understanding cold sensation and predicting the impacts of climate warming. In previous work, I cataloged the TRP family of ion channels in notothenioids and identified candidate thermosensitive TRPs through a combination of transcriptomics and evolutionary dynamics analyses [14]. This work found that *TRPV1a* and *TRPM4* were enriched in the sensory nerves of the Antarctic spiny plunderfish *Harpagifer antarcticus* relative to the whole brain and showed evidence of intensified selection in the Antarctic clade of notothenioids compared to other fishes. I discovered evidence of at least four independent duplications of *TRPA1b* within the Antarctic notothenioid clade, which was also under intensified selection. These findings identified *TRPV1a*, *TRPM4* and *TRPA1b* as the top candidates for thermosensors in these fishes.

Ancestrally, teleost fishes have two paralogs each of *TRPV1* and *TRPA1* probably from the teleost whole genome duplication but *TRPA1a* was lost around the evolution of Neoteleostei and so it is not found in more derived teleost groups such as gadiforms and perciforms, including notothenioids [14]. Studies on TRP channel function in fishes have focused on zebrafish (*Danio rerio*; e.g. [22,23]) and found that both *TRPV1a* and *TRPA1b* are expressed in sensory neurons in zebrafish larvae [24,25]. These studies found knockdowns of *TRPV1a*, but not *TRPA1b*, modulate thermal preference behaviour of larval zebrafish. *In vitro*, TRPV1a from zebrafish and the salmon *Onchorhynchus masou ishikawae* is heat activated with a threshold near 28°C. Modulation of TRPV1 activity shifts temperature preference behaviours in these fishes [24,26]. *TRPV1* expression appears to be regulated by behavioural fever in Atlantic salmon *Salmo salar*, a behaviour in which the fish seeks warmer temperatures to induce fever after an immune challenge [27]. Zebrafish TRPA1b *in vitro* is both cold and heat activated with an average cold activation threshold of 11°C and no distinct warm threshold, however, knockouts did not change escape behaviour between 5 and 40°C [25,28]. TRPA1b from *Takifugu rubipes* had similar behaviour with a cold activation threshold of 8°C while TRPA1b from medaka (*Oryzia latipes*) is only heat activated *in vitro* at temperatures greater than 25°C with no obvious threshold [29,30]. Finally, motor responses to temperature stimuli in zebrafish larvae can be modelled entirely from sensory nerve activity with two cell types, one that is heat activated and the other heat inhibited which implicates a simple peripheral thermosensory mechanism [31].

In this study, I sought to characterize TRPA1b, TRPM4 and TRPV1a from cryonotothenioid fishes using oocyte electrophysiology. I found that TRPA1b and TRPV1a were both heat-activated, but to my surprise, with an activation threshold at least 20°C higher than expected. I hypothesized that specific conditions in the cellular environment of cryonotothenioids shift the thermal sensitivity range of the channel to be relevant for the Southern Ocean. I tested changes in osmolytes, pH, oxidation, phosphorylation, lipids and the presence of accessory proteins and found that altering the lipid membrane through cholesterol reduction in combination with oxidation was able to decrease the activation threshold close to the expected temperature range. I conclude lipid regulation, possibly via changes in lipid raft integrity, may be a mechanism of shifting TRP channel activation thresholds to ecologically relevant temperatures for these fishes.

## 2. Results and discussion

### 2.1. *Gymnodraco acuticeps* TRPA1b

**Figure 1.**
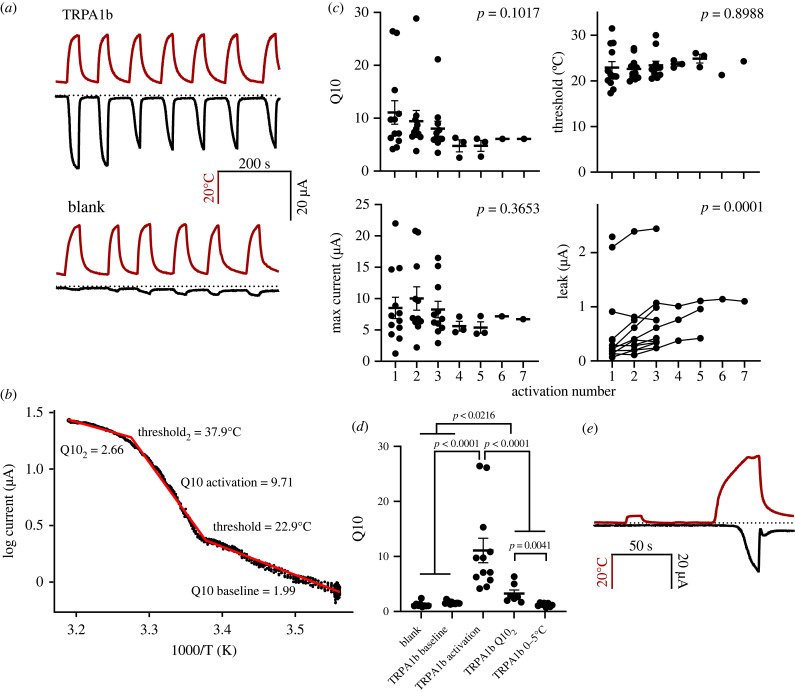
Electrophysiological characterization of TRPA1b from *G. acuticeps*. (*a*) Example traces from TRPA1b injected oocytes (top) and water injected control oocytes (bottom) showing the temperature stimulus in the red trace and the current response in black, dotted line indicates 0 for both *y*-axes. (*b*) Example van't Hoff plot of TRPA1b with a three segment fit to the data in red. (*c*) Q10, threshold, maximum current and leak values for TRPA1b across multiple temperature activations; *p*-values indicate the significance of the effect of the activation number on the dependent variable. (*d*) Q10 values for the water-injected control oocytes, baseline Q10 of TRPA1b injected cells, the Q10 of activation, Q10_2_ or the Q10 above the second threshold, and Q10 of TRPA1b injected cells between 0 and 5°C. (*e*) Example trace of low temperature experiments with temperature stimulus in red and current response in black, dotted line indicates 0 for both *y*-axes.

Oocytes injected with *TRPA1b* mRNA from *Gymnodraco acuticeps* showed robust temperature activation *in vitro* that was not evident in water-injected oocytes (figure 1*a*). Van't Hoff plots of the temperature-activated currents had an average Q10 of 11.09 ± 2.24 and threshold of 22.91 ± 1.31°C (*n* = 12; figure 1). At temperatures lower than the threshold, Q10 was 1.59 ± 0.074, which did not differ from Q10 of water injected controls and reflects the temperature dependence of the endogenous and leak currents from the oocytes (1.24 ± 0.157; n = 9; *p* = 0.3220). For most curves, a second breakpoint at 34.46 ± 0.91°C could be determined; at temperatures above this second threshold Q10 was reduced to 3.26 ± 0.66. This second breakpoint probably arises from channel open probability reaching a maximal value, the Q10 at temperatures above this breakpoint reflects cation diffusion through the open channel pore. Each experiment cycled between low and high temperatures multiple times. With multiple stimulations with heat, the threshold of the TRPA1b channel activation remained constant (*p* = 0.8988) as did the Q10 (*p* = 0.1017). Maximum current was measured as the current at 35°C minus the leak current and this was also constant across multiple activations (*p* = 0.3653). Leak currents measured at sub-threshold temperatures in TRPA1b-expressing oocytes started to increase after the third activation (*p* = 0.0001), but this was not different in water injected controls (*p* = 0.0824). I observed a small but insignificant decrease in threshold with increasing concentrations of injected RNA (slope = −0.098; intercept = 31°C; *p* = 0.1372). I did not observe an increase in current from TRPA1b-expressing oocytes with the application of 100 µM 2-aminoethoxydiphenyl borate (2-APB, data not shown). I also measured current–voltage relationships and found that TRPA1b had increased current with voltage steps at temperatures above 25°C with an EC_50_ of −17.22 ± 5.17 mV (electronic supplementary material, figure S1A).

### 2.2. *Notothenia coriiceps* TRPV1a

**Figure 2.**
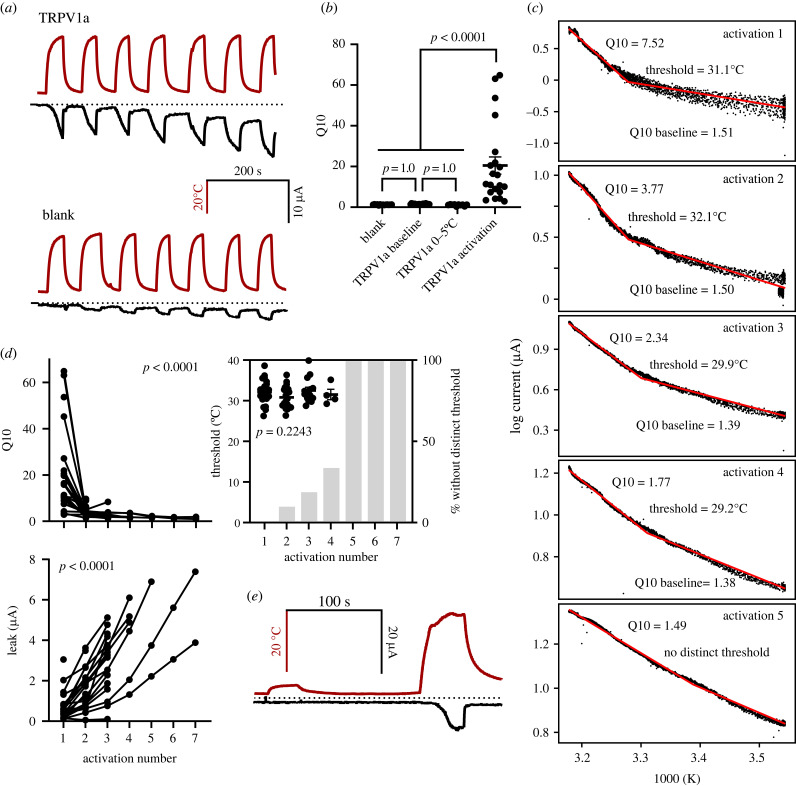
Electrophysiological characterization of TRPV1a from *N. coriiceps.* (*a*) Example traces from TRPV1a injected oocytes (top) and water injected controls (bottom) showing the temperature stimulus in red and the current response in black, dotted line indicates 0 for both *y*-axes. (*b*) Q10 values for water injected controls compared to baseline, Q10 of TRPV1a cells between 0 and 5°C, and activation Q10 values for TRPV1a injected oocytes. (*c*) Example van't Hoff plots for one oocyte across multiple temperature activations showing the fits to the data in red and calculated dependent variables. Note that the relationship between log current and inverse temperature becomes more linear with multiple activations. (*d*) Calculated Q10, threshold and leak values for TRPV1a injected oocytes across multiple activations; *p*-values indicate the significance of the effect of activation number on the dependent variable. Black lines connect values from the same oocytes across activations. Grey bars in the threshold plot show the percentage of cells tested that showed no distinct threshold (such as activation 5 from figure 2*c*). (*e*) Example trace of low temperature experiments with temperature stimulus in red and current response in black, dotted line indicates 0 for both *y*-axes.

In oocytes expressing the TRPV1a channel from *Notothenia coriiceps*, I observed large increases in current when temperature was increased (figure 2*a*), which had Q10 values on initial activation of 20.48 ± 2.42 and thresholds of 32.11 ± 0.637°C (*n* = 21; figure 2*b*). This was significantly higher than the Q10 measured below the threshold (‘baseline Q10': 1.43 ± 0.041; *n* = 23; *p* < 0.0001). The baseline Q10 did not differ from the Q10 of water injected cells (1.23 ± 0.035; *n* = 14; *p* = 1.0). For TRPV1a, I did not observe a second breakpoint at higher temperatures, indicating that channels did not reach maximal activation over the range of temperatures studied (approx. 0–40°C). Upon subsequent stimulations with heat, the Q10 of channel activation decreased (activation 2: 3.95 ± 0.445, *n* = 22; activation 3: 2.70 ± 0.418, *n* = 16; activation 4: 2.31 ± 0.396, *n* = 6; activation 5: 1.92 ± 0.225, *n* = 3; activation 6: 1.49 ± 0.265, *n* = 2; activation 7: 1.45 ± 0.464, *n* = 2; *p* = 0.00001). By contrast, thresholds did not change with multiple activations (*p* = 0.2243). However, the proportion of curves that showed no distinguishable threshold increased such that by the fifth activation, no cells had a distinct or measurable activation threshold (figure 2*c*). There was no relationship between RNA concentration injected and threshold (slope = 0.0075; intercept = 31.3°C; *p* = 0.5891). Baseline currents at temperatures below the threshold increased significantly with multiple activations (*p* < 0.0001) and significantly more than in water injected control cells (*n* = 13 controls; *p* = 0.03), suggesting that the baseline open probability of TRPV1b channels increases with repeated stimulations with heat. Previous work on rat TRPV1 found the same behaviour with repeated temperature activation and the authors posit this is due to a partial unfolding meaning the channel is unable to continue translating heat into a conformational change [32]. Whether this occurs *in vivo* is unknown. I was not able to measure reliable conductance–voltage relationships for TRPV1a up to the maximum test voltage of 50 mV, some increase in current with voltage could be seen for this channel at 35°C, but not other temperatures (electronic supplementary material, figure S1C).

### 2.3. TRPM4 and cold activation

I was never able to measure temperature-activated currents from *Harpagifer antarcticus* TRPM4, despite using similar concentrations of RNA, the same incubation period and the same oocyte batches as the other channels. I also never measured cold activated currents from any channel, even when cells were held at high temperatures before clamping (data not shown).

### 2.4. Altering cellular conditions

The data so far have established TRPA1b from *Gymnodraco acuticeps* and TRPV1a from *Notothenia coriiceps* are activated by increased heat *in vitro*, but, surprisingly, both channels activate at temperatures at least 20°C warmer than expected. No high Q10 currents from either channel were seen between 0 and 5°C (figure 1*d*,*e*; figure 2*b*,*e*). To my knowledge, this is the first characterization of a TRPV1 channel that shows temperature activation in a temperature range completely irrelevant for the organism in their native habitat. Given these findings, I propose three alternative hypotheses for molecular mechanisms of temperature sensation in cryonotothenioid fishes: (1) another molecular mechanism entirely underlies peripheral temperature sensation in these fishes; (2) cryonotothenioids lack the molecular machinery to sense temperature changes in the range of their native habitats; (3) conditions in the cellular environment of cryonotothenioids shift the thermal sensitivity of the channel to be relevant for the Southern Ocean. A cascade of physiological adaptations underlies the notothenioids' ability to diversify successfully at temperatures around the freezing point of their body fluids. To test hypothesis 3, I tested several physiological characteristics of Antarctic notothenioids for their effect on TRP channel function. Hypotheses 1 and 2 are also discussed but not tested here.

### 2.5. Antarctic notothenioids have double the osmolality of a typical teleost

**Figure 3.**
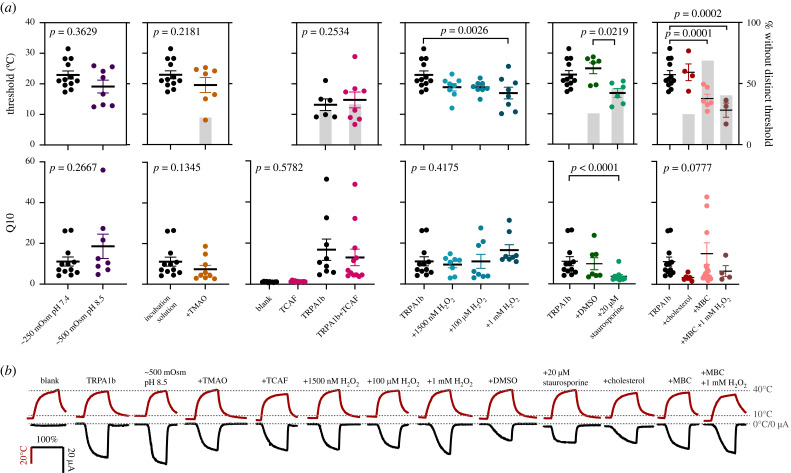
Oxidation, reduced phosphorylation and reduced membrane cholesterol lower the threshold of TRPA1b. (*a*) Threshold (top row) and Q10 (middle row) values for TRPA1b comparing control conditions (black) with various treatments to alter cellular conditions (colours). Grey bars indicate the percentage of cells tested that showed no distinct threshold of activation. *p*-values refer to the significance of the treatment conditions on either threshold or Q10, pairwise statistical comparisons are specifically indicated if significant. (*b*) Example curves for each of the conditions shown, red curves are temperature and black are current. For better visual comparison, time is displayed as time/time to peak temperature as a percentage.

**Figure 4.**
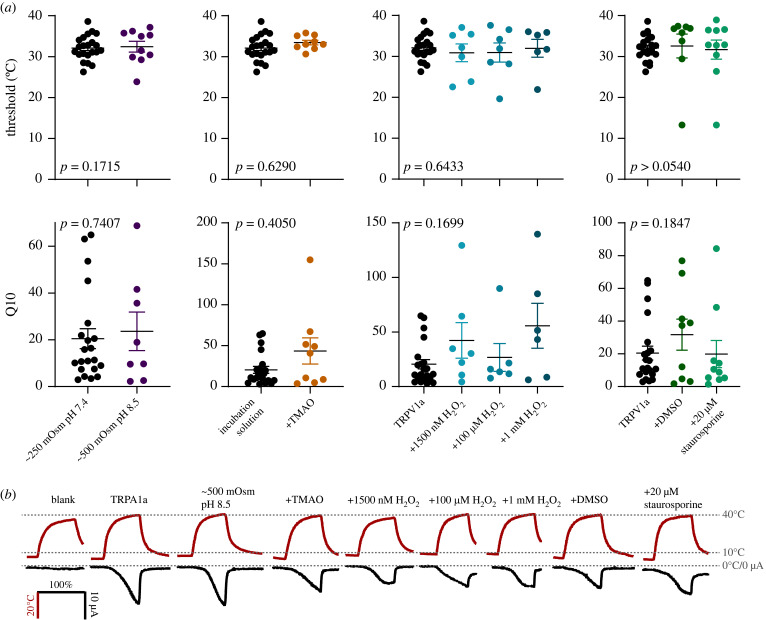
No conditions tested altered threshold of TRPV1a activation. (*a*) Threshold (top row) and Q10 (middle row) values for TRPV1a comparing control conditions (black) with various treatments to alter cellular conditions (colours). *p*-values refer to the significance of the treatment conditions on either threshold or Q10. (*b*) Example curves for each of the conditions shown, red curves are temperature and black are current. For better visual comparison, time is displayed as time/time to peak temperature as a percentage.

Antarctic notothenioid fishes famously express antifreeze proteins that drop the freezing point of their body fluids but even without these proteins their body fluids have freezing points of about −1°C due to an osmolality double that of a typical teleost (approx. 500 mOsm [33,34]). Further, blood pH is 8.2–8.4 [35]. Several TRP channels are known to be modulated by changes in osmolality and pH [36]. I doubled the osmolarity of the external solution and increased the pH to 8.5, making the external solution a ‘notothenioid Ringer's' and found no difference in threshold (TRPA1b: *n* = 8; *p* = 0.3629; TRPV1a: *n* = 9; *p* = 0.1715) or Q10 (TRPA1b: *n* = 8; *p* = 0.2667; TRPV1a: *n* = 8; *p* = 0.7407; figures 3 and 4). There was a significant effect of the higher osmolarity condition increasing leak after channels were activated multiple times, due to a stronger concentration gradient (driving force) across the membrane (TRPA1b: *n* = 8; *p* = 0.0389; TRPV1a: *n* = 9; *p* = 0.0039: electronic supplementary material, figure S2). This also increased maximum current for TRPA1b (*n* = 8; *p* = 0.0093).

### 2.6. Compatible osmolytes or accessory proteins could help stabilize cold adapted proteins

Proteins can denature at cold temperatures due to decreasing entropy resulting in the ordering of water molecules along the protein chain [37]. Cold adapted proteins are less stable at all temperatures due to a reduction in the enthalpy of unfolding because of reduced heat capacity [38]. The result, for notothenioids, is frequent degradation of proteins as demonstrated by high levels of ubiquitination [39]. Proteins can be stabilized through ‘molecular crowding' or through the presence of compatible solutes [40,41]. Notothenioid fishes have high levels of the compatible solute trimethylamine oxide (TMAO) in their tissues and constitutively high expression of chaperone proteins, both of which may help to stabilize proteins [42–44]. I hypothesized that stabilizing the channels at low temperatures by crowding or compatible solutes could modulate gating temperature of these TRP channels. To test this, I replaced 15% of the total osmolality of the incubation solution with TMAO to mimic the concentrations in notothenioid tissues.

There was no significant effect of TMAO incubation on threshold (TRPA1b: *n* = 7; *p* = 0.2181; TRPV1a: *n* = 9; *p* = 0.6290) or Q10 for either of the channels (TRPA1b: *n* = 9; *p* = 0.1345; TRPV1a: *n* = 8; *p* = 0.4050; figures 3 and 4). With TMAO treatment, about 25% of oocytes expressing the TRPA1b channel maintained Q10 but showed no distinct threshold of activation compared to 0% for the control, suggesting some channels may have already been open at the holding temperatures (6.0 ± 0.1°C). Interestingly, TMAO had the opposite effect on TRPV1a where all the TMAO treated cells had a measurable threshold of activation even at the third activation. The presence of TMAO significantly reduced leak after the first activation of the channel in TRPV1a (*n* = 9; *p* = 0.0392; electronic supplementary material, figure S3) but not TRPA1b (*n* = 7; *p* = 0.6065). The TMAO treatment also increased maximal currents for TRPA1b compared to untreated TRPA1b controls (*n* = 9; *p* = 0.0083).

Given that incubation with TMAO increased the proportion of TRPA1b oocytes that had no distinct threshold without reducing the Q10, and also increased the maximum current, it may have increased channel expression possibly through stabilizing the proteins in the membrane [45,46]. The opposite effect of TMAO on the threshold of TRPV1a may also be due to stabilization, in this case slowing the partial denaturation that occurs with multiple heat activations, as previously discussed. I conclude TMAO is likely not a major determinant of activation threshold but may help in general protein stabilization.

### 2.7. TRP channel associated factors are found in notothenioids

I also tested co-injection of TRPA1b with a chaperone protein. Very little work has examined accessory proteins of temperature activated TRP channels, yet it is thought that most TRP channels form supramolecular complexes [47]. Gkika *et al*. [48] found about 200 peptides that interacted with either the C or N-terminal of TRPM8 from mice, nearly 70 of which are unnamed. They investigated two human homologs of these proteins and named them TRP channel associated factors (TCAFs). I used the TCAF sequences from the 2015 study to look for homologs in notothenioid fishes and identified four genes that grouped with TCAF from *Danio rerio* in a gene tree. One of these genes I found was enriched in neural tissue compared to non-neural tissue (electronic supplementary material, figure S4). I found the homologs of that gene in *G. acuticeps* and co-injected this TCAF with TRPA1b. Unexpectedly, TRPA1b control oocytes for these experiments had lower thresholds and a higher proportion of oocytes with no distinct threshold compared to other experiments (figure 3). Given these controls, I found no difference with co-injection of TCAF in threshold (*n* = 12; *p* = 0.2534), Q10 (*p* = 0.5782), leak (*p* = 0.0688; electronic supplementary material, figure S5), or maximum current (*p* = 1.0). There was no difference in Q10 (*n* = 10; *p* = 1.0) or maximum current (*p* = 1.0) between cells injected with the TCAF mRNA alone compared to water injected controls. I also found the same homolog in *N. coriiceps* but positive control injections were unsuccessful for these experiments.

It is possible *TCAF* did not express although it was optimized for *Xenopus* expression under the same promotor as *TRPA1b*; it is also possible that this TCAF protein does not interact with TRPA1b, leaving its purpose in neural tissues in fishes to be explained. Human TCAF modulated the temperature-dependent activity of TRPM8, which is a mild cool sensor in mammals. Fishes lack *TRPM8* [49].

### 2.8. Oxidation lowers the threshold of TRPA1b

Oxygen is more soluble in cold water and cryonotothenioids have high levels of oxidative stress, despite elevated expression of antioxidants [50,51]. Both TRPA1 and TRPV1 from other species can be activated by oxidants [28,52–55]. I tested TRPA1b and TRPV1a with 1500 nM, 100 µM and 1 mM H_2_O_2_ applied extracellularly. The 1500 nM H_2_O_2_ was selected as a starting point as this is estimated to be the maximum concentration of H_2_O_2_ when it snows on the Southern Ocean [56]. I found that 1 mM extracellular H_2_O_2_ significantly reduced the threshold of activation for TRPA1b, to an average threshold of 16.97 ± 1.92°C, a difference of about 6°C (*n* = 8; *p* = 0.0026; figure 3). The lower concentrations reduced the threshold non-significantly by about 4°C. H_2_O_2_ at any concentration did not change Q10 of activation (*n* = 8 for all H_2_O_2_ conditions; *p* = 0.4175), leak (*p* = 0.8653; electronic supplementary material, figure S6), nor maximum current for TRPA1b (*p* = 0.077). For TRPV1a, there was no difference at any concentration of H_2_O_2_ in threshold (*p* = 0.6433; figure 4), Q10 (*p* = 0.1699), or leak (*p* = 0.1351; electronic supplementary material, figure S6).

I conclude that extracellular redox state may be important for determining temperature sensitivity thresholds in TRPA1b but not TRPV1a. Further work on intracellular application of oxidants is needed for TRPV1a. Mutation of cysteines and methionines would be able to determine whether the TRPA1b channel is being oxidized directly or if the channel is being modulated by indirect effects, such as lipid oxidation. Methionine is overrepresented in the proteins of Antarctic notothenioids compared to temperate fishes which may make their proteins generally more sensitive to redox state [57].

### 2.9. Inhibiting phosphorylation shifts the voltage sensitivity of TRPA1b

Reduced phosphorylation through application of the kinase inhibitor staurosporine has been shown to shift thermal threshold of mammalian cool sensor TRPM8, increasing responses to cold stimuli through a left shift in voltage activation curve combined with an increase in the number of channels in the membrane [58]. I tested TRPA1b with staurosporine dissolved in DMSO as well as a DMSO control. There was a significant decrease in threshold for TRPA1b with staurosporine treatment compared to DMSO alone (to an average of 16.9 ± 1.46°C; *n* = 6 for both conditions; *p* = 0.0219) but not compared to untreated control conditions (*p* = 0.0860; figure 3). Out of 11 TRPA1b-injected oocytes treated with staurosporine, 5 (46%) showed no clear threshold at all, compared to 25% of oocytes (2/8) treated with DMSO and 0/12 control oocytes. I tested whether this change in threshold was due to a shift in the current–voltage relationship and found a significant decrease in V_50_ with staurosporine treatment (average with staurosporine at 25°C was −35.8 ± 6.24 mV; *n* = 7; *p* = 0.0458; electronic supplementary material, figure S1B). This suggests that phosphorylation might be a more general regulator of voltage sensitivity of TRP channels.

However, staurosporine, but not DMSO, significantly decreased Q10 of TRPA1b from 11.09 ± 2.24 to 3.72 ± 0.85 (*p* < 0.0001), essentially eliminating the significant temperature dependence. There was no change in baseline Q10 (*p* = 0.4627). Leak increased significantly after the first activation with staurosporine treatment (*p* = 0.0002; electronic supplementary material, figure S7) and maximal current was significantly higher (*p* = 0.0060). For TRPV1a, there was a significant increase in threshold with either staurosporine or DMSO treatment (*n* = 9 for staurosporine; *n* = 7 for DMSO; *p* = 0.0044; figure 4), however, this was not significant at the level of post hoc comparisons (*p* ≥ 0.0540). There was no difference in TRPV1a Q10 with staurosporine or DMSO (*n* = 10 for staurosporine; *n* = 9 for DMSO; *p* = 0.1847). Leak increased significantly with staurosporine specifically at the third activation (*n* = 10; *p* = 0.0010; electronic supplementary material, figure S7) but baseline Q10 did not (*n* = 10; *p* = 0.1536).

### 2.10. Membrane lipids help determine temperature activation threshold

Some TRP channels, including TRPA1 and TRPV1, are associated with lipid rafts in the synaptic membrane and their activity is modulated by lipid raft integrity [59,60]. Lipid rafts are characterized by enrichment in sphingolipids and cholesterol, act to colocalize signalling molecules, and can be disrupted by incubation with methyl-β-cyclodextrin (MBC) which depletes cholesterol [61–63]. Interestingly, notothenioids are specifically depleted in sphingolipids and lack expression of sphingolipid metabolic genes but it is unknown how this might affect lipid rafts in these fishes [64,65]. Adaptation to cold temperatures often results in adjustment of lipid structure or content in cell membranes to maintain homeoviscousity (but see [66]). In brain synaptic membranes, cryonotothenioids have only partially compensated for loss in viscosity due to cold. In other words, notothenioid brain synaptic membranes are less viscous at their habitat temperature compared to other species [67]. Furthermore, cryonotothenioid brain membranes did not show any homeoviscous compensation in response to 5°C acclimation over six weeks, while other tissues did (i.e. they became more fluid with rising temperatures and stayed that way; [68]). I hypothesized the interaction of membrane lipids and TRP channels is important for determining the temperature activation range of the channels and tested TRPA1b with the addition of cholesterol-enriched liposomes and the depletion of cholesterol through incubation with MBC.

Threshold of TRPA1b was significantly reduced with MBC treatment by about 8°C to an average threshold of 15.1 ± 1.41°C (*n* = 19; *p* = 0.0001; figure 3). This difference was only significant on the initial activation (electronic supplementary material, figure S8; *p* = 0.2168 on second activation). Out of 19 oocytes tested with MBC, 14 (74%) had no measurable threshold on initial activation but Q10 was not different (*n* = 19; *p* = 1.0) from untreated TRPA1b controls, indicating the channels might already be open at the holding temperatures (6.6 ± 0.6°C). Leak (*n* = 19; *p* = 0.0194), current at 10°C (*n* = 19; *p* = 0.0230), and overall maximum current was significantly higher in MBC treated oocytes compared to untreated TRPA1b controls (*n* = 19; *p* = 0.0001; electronic supplementary material, figure S8).

I also enriched the oocyte membrane with cholesterol. Cholesterol enrichment did not change threshold (*n* = 6; *p* = 1.0; electronic supplementary material, figure S8), Q10 (*n* = 6; *p* = 0.1240), leak (*n* = 6; *p* = 0.0731), nor maximum current (*n* = 6; *p* = 1.0). I interpret the lack of effect of cholesterol enrichment as support for a mechanism of lipid raft disruption for the effect of MBC, rather than a direct relationship with membrane fluidity. However, efficacy of cholesterol enrichment was not tested.

With MBC and cholesterol treatments, Q10 did not change in water injected controls (*p* = 0.3957; average of 1.40 ± 0.32 for adding cholesterol; *n* = 8; average of 1.35 ± 0.11 for MBC; *n* = 8). MBC treatment, but not cholesterol, increased leak specifically at the third activation in water injected controls (*n* = 6; *p* = 0.0082) and maximum current for the first activation only (*n* = 8; *p* = 0.0120).

These results suggest lipid raft integrity, and not membrane fluidity, may help shift the activation threshold of temperature activated channels. This proposed mechanism might globally effect ion channel function in cryonotothenioids and explain the loss of sphingolipid metabolism. Further work examining lipid raft structure in native and heterologous systems and across other channels is needed to understand how channels can shift to function at low temperatures.

### 2.11. Additive effects

To investigate whether multiple conditions might have additive or other synergistic effects on TRPA1b properties, MBC treated oocytes were tested also adding 1 mM H_2_O_2_ to the external solution. Threshold for MBC and oxidant treated oocytes was not different to MBC treatment alone (*n* = 5; *p* = 1.0; figure 3) although the average threshold was trending lower (average 11.28 ± 2.34°C). However, no distinct thresholds could be measured in 40% of these oocytes from the holding temperature of 2.6 ± 1.5°C yet Q10 was maintained (*n* = 5; *p* = 1.0) suggesting that these channels may be activating in the expected temperature range. Leak (*n* = 5; *p* = 1.0) and maximum current (n = 5; *p* = 1.0) were not different for the MBC + H_2_O_2_ treated oocytes compared to MBC treatment alone.

### 2.12. Hypothesis 3: summary

I aimed to identify cellular conditions that might shift temperature dependent activity of TRPA1b and TRPV1a from Antarctic notothenioid fishes to an ecologically relevant range. I found no tested experimental condition lowered the threshold of *Notothenia coriiceps* TRPV1a. I found reduced cholesterol lowered the threshold of *Gymnodraco acuticeps* TRPA1b activation to about 15°C, still out of the range of temperatures in the Southern Ocean. However, a large proportion (74%) of cells had no distinct threshold yet maintained their Q10, indicating that the channels were already open at the holding temperatures (6.6 ± 0.6°C). This could be confirmed in future studies with use of a blocker. In combination with extracellular oxidation, threshold was non-significantly lower (approx. 11°C) on average and 40% of these oocytes had no distinct threshold from a holding temperature of 2.6 ± 1.5°C. I conclude that TRPA1b shows temperature-dependent activation in the range of noxious temperatures for cryonotothenioids but the intrinsic activation range of the protein requires modulation, possibly from membrane lipids and oxidation. I also found reduced phosphorylation lowered the threshold by shifting the voltage sensitivity of TRPA1b to lower voltage potentials but eliminated the strong temperature dependence of channel opening. I identified *TCAF* genes in notothenioid fishes and found one that had enriched expression in neural tissues, however, co-injection of a *TCAF* gene did not change temperature activation properties of TRPA1b.

For this study, two-electrode voltage clamp in *Xenopus* oocytes was used, however, there are drawbacks to heterologous expression systems. In this case, the use of *Xenopus* oocytes made sense as they are incubated at relatively cool temperatures compared to other systems. However, they require minimum temperatures around 15°C for functional translation [69]. I used 14°C but this may affect the properties of the proteins. It is possible that expression at their native temperature may alter intrinsic channel properties. Future studies might develop cryonotothenioid cell systems to test channels at more realistic temperatures.

### 2.13. Hypothesis 2: do Antarctic notothenioids even sense temperature?

Given the extreme stenothermy of the Southern Ocean it would be reasonable to hypothesize that the sense of temperature has been lost in cryonotothenioid fishes. Limited data exist on temperature preference in these fishes; Crawshaw & Hammel [70] found that *N. coriiceps* and the icefish *Chaenocephalus aceratus* escaped temperatures above 5°C before deep body temperature warmed, indicating the use of peripheral thermosensors. Robinson [71] found no temperature preference behaviour for *Pagothenia borchgrevinki* between 0 and 4.5°C. Fanta *et al*. [72] noted stress behaviours during warming beginning around 4–5°C for three Antarctic species. Notothenioid larvae show species-specific temperature distributions, on average 40% of the variation in these distributions can be explained by temperature [73]. At least one notothenioid species *Pleurogramma antarctica*, a key prey item for penguins and seals, is being displaced in the more northern parts of their range, probably by the upwelling of warm (approx. 2°C) circumpolar deep water [74]. Given these data and that temperatures as low as 6°C can be lethal for some species [75], I expect that cryonotothenioids maintain a mechanism for sensing temperatures greater than 5°C even if they do not generally experience those temperatures in their current thermal environment. However, more robust data on temperature preference in these fishes would better test the hypothesis of lost temperature sensation.

### 2.14. Hypothesis 1: data are available for investigation into other channels and tissues

In identifying candidate thermosensors in notothenioids, I focused on TRP channels expressed in the sensory nerves. Several other channels have been proposed as possible mechanisms of temperature sensation in the cold including potassium, sodium, and P2X receptor channels (recently reviewed in [76]). Data from mammals indicates primary keratinocytes amplify thermal signals via an unknown molecular mechanism, potentially modulating the activity of sensory afferents [77]. It is possible a similar mechanism exists in fish, and skin transcriptomes for notothenioids at various temperatures are publicly available (e.g. [78]). Fishes interface with their environment through the gills and *TRPV1* is upregulated in the gill during summer in Atlantic salmon, indicating that the gill could be an interesting site for further investigation [79]. Future studies could compare increasingly available sequence data across species and tissues to identify new candidate channels [80].

### 2.15. Applications

Molecular thermosensors that function reliably have industrial, medical and research applications [81–83]. Here, I describe TRPA1b from *G. acuticeps*, a channel with robust temperature-dependent activation that occurs intrinsically just above room temperature with consistent behaviour across multiple activations. While the wild-type channel is modulated by lipids, oxidation and phosphorylation, it is possible that sites involved in this response could be mutated to create a channel that responds only to temperature. Indeed, I found this channel already shows no activation from 2-APB, a common agonist of TRP channels [84,85].

### 2.16. Conclusion

I conclude TRPA1b is likely to be a functional temperature sensor in cryonotothenioid fishes but requires modifications to function at ecologically relevant temperatures. I propose lipid raft disruption as a potential global mechanism for shifting ion channel function. These hypotheses rely on interpretations of the data that could be confirmed through further testing, such as application of a blocker to test whether channels are actually open at the holding temperature when no threshold is seen but Q10 is maintained. TRPV1a appears to have temperature dependent activity only in the range of temperate teleost channels, and therefore is likely not a functional temperature sensor in cryonotothenioid fishes.

## 3. Methods

### 3.1. Cloning and RNA synthesis

*Gymnodraco acuticeps TRPA1b* was obtained from the transcriptome shotgun assembly (GGFR00000000) sequenced from embryonic tissue, adult brain, gill, liver, and spleen and pooled. This sequence is nearly identical to NCBI reference sequence accession XM_034216404.1, with one substitution: Y298I. *Notothenia coriiceps TRPV1a* (XM_010792623.1) was predicted from the genome assembly [86]. *Harpagifer antarcticus TRPM4* was obtained from a transcriptome pooled from juvenile brain, heart, white muscle, liver, skin and kidney (PRJEB26835). These species were selected due to availability of the most complete sequence for each gene. Sequences can be found in the supplemental material. GenScript (Piscataway, NJ, USA) optimized each gene for expression in both *Xenopus* and mammalian systems and synthesized and cloned the genes into pMO, a modified *Xenopus*/mammalian vector kindly provided by Dr Elena Gracheva (Yale University). RNA was synthesized *in vitro* using the mMessage mMachine T7 transcription kit (ThermoFisher Scientific), concentration was measured using an ND-1000 spectrophotometer (NanoDrop Technologies, Wilmington, DE, USA), and RNA quality was confirmed using either electrophoresis (TapeStation, Agilent, Santa Clara, CA, USA) or fluorometry (Qubit, ThermoFisher Scientific, Waltham, MA, USA). RNA stocks were stored at −80°C and aliquots were stored at −20°C.

### 3.2. Oocyte injection

Mature (9 + cm) *Xenopus laevis* females were obtained from Nasco, WI (Item #LM00535MX) and housed in the University of Texas animal facility. Frogs were anaesthetized using tricaine and oocytes were surgically removed in accordance with NIH guidelines and IACUC approved protocol (AUP-2015–00205) at the University of Texas at Austin. Oocytes were stored away from light for 2–10 h at room temperature in an isotonic solution. To isolate individual oocytes the thecal and epithelial layers were manually removed with foreceps in a solution containing 108 mM NaCl, 1 mM EDTA, 2 mM KCl and 10 mM HEPES. The follicular layer was removed by agitation for 10 min in a solution containing 83 mM NaCl, 2 mM KCl, 1 mM MgCl_2_, 5 mM HEPES and 0.5 mg ml^−1^ collagenase from *Clostridium histolyticum*. Defolliculated oocytes were injected with mRNA suspended in water (total of 50 nl) using a microinjector (Drummond Scientific Co., PA, USA). For *TRPM4*, 100 ng of RNA were injected per oocyte, for *TRPA1b* between 78 and 100 ng per oocyte, for *TRPV1a* between 21 and 100 ng were injected per oocyte. Oocytes were incubated for three days; *TRPV1a* and *TRPM4* injected oocytes were incubated at 14°C and *TRPA1b* oocytes were incubated at 14°C for the first 24 h and then moved to 9°C for the subsequent 2 days. For all conditions tested, oocytes injected with 50 nl of water without RNA and incubated at the same temperature were used as controls.

### 3.3. Temperature activation experiments

On day 3, oocytes were removed from the incubator and kept on ice until use. Individual oocytes were placed on a low volume oocyte perfusion chamber (AutoMate Scientific, Berkeley, CA) and perfused with a solution held between 5–9°C containing 115 mM NaCl, 1.5 mM KCl, 10 mM HEPES, 1 mM MgCl_2_, 1 mM EGTA, adjusted to pH 7.4 with NaOH at a flow rate of 1.1 ml min^−1^. A two-electrode voltage clamp was used to hold voltage constant at −60 mV and measure current responses; electrodes were filled with 3 M KOAc and 15 mM KCl. Chlorided reference electrodes were placed in an adjoining chamber connected by an agar bridge and filled with 3 M KCl. Bath temperature was controlled by two inline solution heater/coolers and a dual channel bipolar temperature controller (Warner Instruments, Holliston, MA, USA) and recorded with a thermistor placed 1–2 mm downstream of the oocyte. Excess heat generated from the Peltier elements was removed by the liquid cooling system (Warner Instruments, Holliston, MA, USA) allowing a temperature range of 5–45°C at the oocyte. Temperature, current and voltage were recorded gap-free with pClamp software (version pre-9.0, Molecular Devices, San Jose, CA, USA) at a rate of 2 kHz. Temperature sensitive current was measured by bringing the oocyte between the cool holding temperature (less than 10°C) to 40°C at least three times in rapid succession (one heating/cooling cycle lasted about 1 min). Current–voltage relationships were investigating using a voltage step protocol with 10 mV voltage steps between −90 and +40 mV lasting 100 ms followed by a 100 ms −50 mV tail pulse from a −90 mV holding potential.

To test temperatures less than 5°C, the perfusion chamber was attached to a glazed clay tile adhered to a cooling plate with thermal paste (Stir Kool Model SK12, Thermoelectrics Unlimited, Wilmington, DE, USA). With assistance of the cooling plate, the perfusate was cooled to between 0 and 1°C, or lower if possible, and then a 5°C solution was applied to the oocyte for 10–20 s after which the oocyte was rapidly cooled to 0°C again. The flow rate for these experiments was 2 ml min^−1^ Expression was verified immediately by two heating cycles between 5 and 40°C.

### 3.4. Manipulations

For high osmolarity experiments, molarity of the external solution was doubled and pH was adjusted to 8.5. To test TMAO, 15% of the osmolarity of incubation solution was replaced by TMAO by displacing 15 mM NaCl with 30 mM TMAO. Oocytes were incubated in the TMAO incubation solution for 3 days as normal and tested with standard external solution. For oxidation experiments, either 1500 nM, 100 µM or 1 mM H_2_O_2_ were added to external solution. For 2-APB experiments, 100 µM 2-APB was added to external solution and applied for 30 s followed by temperature changes to ensure expression. For phosphorylation experiments, staurosporine was dissolved in DMSO and added to external solution to a final concentration of 20 µM. Oocytes were transferred for one hour either into the staurosporine solution or a control solution with the same volume of DMSO without staurosporine.

For cholesterol addition experiments, I followed the protocol of Slayden and colleagues [87]. Briefly, cholesterol enriched liposomes were prepared using cholesterol, L-α-phosphatidylethanolamine and 1-palmitoyl-2-oleoyl-sn-glycero-3-phospho-l-serine dissolved in chloroform and evaporated under a stream of nitrogen. Lipids were re-suspended in a solution of 150 mM KCl and 10 mM Tris-HEPES at a pH of 7.4 and sonicated gently for 10 min. Oocytes were transferred into this solution and rotated for 10 min on an orbital shaker before testing. For MBC experiments, 50 mM MBC was dissolved into external solution and oocytes were transferred into this solution and kept at room temperature for one hour before testing. MBC experiments were repeated with the addition of 1 mM H_2_O_2_ in the external solution to test for additive effects of manipulations. The efficacy of cholesterol addition or removal from the membrane was not measured.

### 3.5. Accessory proteins

To test the effects of TCAFs, we examined the transcriptome of *Harpagifer antarcticus* (PRJEB26835) for *TCAF* sequences using blastn [88] with sequences from Gkika *et al*. [48] as bait. After recovering the sequences, I confirmed their identity by building a gene tree. I aligned the sequences with MAFFT (version 7.490) [89] and built maximum-likelihood trees with RAxML-ng (version 0.9.0) [90] with default parameters and a GTR substitution model. Sequences that did not group with the other TCAF sequences were eliminated, the sequences were re-aligned, and the tree was built again. Bootstrap values were determined by using the —bsconverge option from RAxML-ng. I examined our 3′-targeted transcriptome data from *H. antarcticus* from a previous study that targeted the expressed genes in the whole brain, trigeminal ganglion, pancreas and liver [14]. I found one of the *TCAF* sequences had expression in the neural tissues but not non-neural tissues (electronic supplementary material, figure S4B). That sequence was used to blast the *G. acuticeps* transcriptome (PRJNA422913) and the *N. coriiceps* (PRJNA268318) genome on NCBI. The sequences that matched were added to the tree to confirm their identity and then optimized for *Xenopus* and mammalian expression, synthesized, and cloned into the pMO vector by Genscript (Piscataway, NJ, USA). RNA was synthesized *in vitro* as described. To conduct these experiments, I injected oocytes with water alone, 25 ng of *TCAF* alone, 69 ng of *TRPA1b* alone, or 69 ng of *TRPA1b* plus 25 ng of *TCAF*. For *TRPV1a*, the same strategy was used but 79 ng of *TRPV1a* RNA was injected. Oocytes were then incubated and tested as described above.

### 3.6. Chemicals

Lipids were obtained from Avanti Polar Lipids (Alabaster, AL, USA). MBC was obtained from MedChem Express (Princeton, NJ, USA). TMAO, cholesterol, staurosporine and hydrogen peroxide were obtained from Sigma Aldrich (St. Louis, MO, USA). All other chemicals were obtained from ThermoFisher Scientific (Waltham, MA, USA).

### 3.7. Data analysis

Data were downsampled from 2 kHz to 100 Hz in Clampfit to reduce noise. Individual response curves were extracted and used to plot the log of the absolute value of current versus the inverse of the temperature in Kelvin in R (version 4.2.1) with ggplot2 (version 3.3.6). Curves were fitted with segmented linear fits using the R package segmented (version 1.6) with either one or two breakpoints (thresholds), depending on the shape of the curve. Q10s were calculated for each segment using the equation

$$Q10=\;\frac{{\mathrm{current}}_{2}^{10/T_{2}-T_{1}}}{{\mathrm{current}}_{1}},$$

where current_2_ is the current value at temperature *T*_2_ and current_1_ is the current value at temperature *T*_1_.

To statistically compare thresholds, Q10s, and other dependent variables while accounting for multiple curves per oocyte I generated linear mixed models and compared main effects of condition, curve number and the interaction, with individual oocytes as a random effect. Using the rstatix package (version 0.7.0), data were examined for normality and extreme outliers were removed; if normality was not achieved, data were log transformed. Models were generated using the nlme package (version 3.1) then the estimated marginal means were compared post hoc with Bonferroni correction using the emmeans package (version 1.8.1).

## Data Availability

All raw data are available at the United States Antarctic Program Data Center (https://doi.org/10.15784/601695) [91]. Supplementary material is available online [92].
